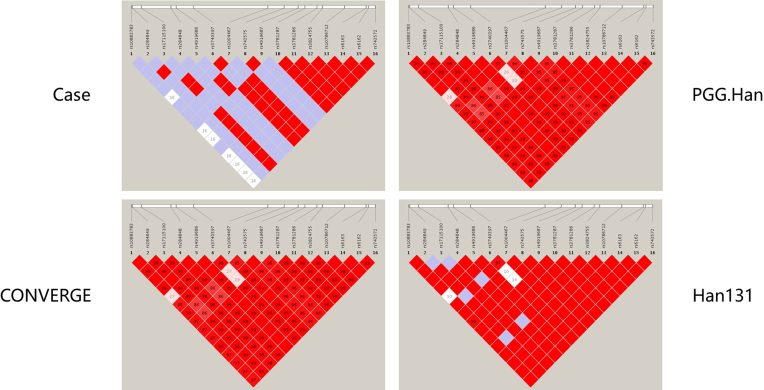# Population-based screening of *CYP17A1* Y329fs mutation carriers in the Han Chinese population

**DOI:** 10.1016/j.gendis.2025.101647

**Published:** 2025-04-15

**Authors:** Bing Han, Xiaoxi Zhang, Chang Liu, Hui Zhu, Wenjiao Zhu, Yue Xu, Fengxue Zhang, Kaiwen Zhang, Shuhua Xu, Jie Qiao

**Affiliations:** a Department of Endocrinology, Shanghai Ninth People's Hospital, Shanghai Jiao Tong University School of Medicine, Shanghai 200011, China; b Key Laboratory of Computational Biology, Shanghai Institute of Nutrition and Health, University of Chinese Academy of Sciences, Chinese Academy of Sciences, Shanghai 200031, China; c Department of Liver Surgery and Transplantation, Liver Cancer Institute, Zhongshan Hospital, Fudan University, Shanghai 200032, China; d State Key Laboratory of Genetic Engineering, Center for Evolutionary Biology, Human Phenome Institute, Collaborative Innovation Center of Genetics and Development, School of Life Sciences, Fudan University, Shanghai 200438, China

17ɑ-hydroxylase/17,20-lyase deficiency (17OHD) was caused by mutations in the *CYP17A1* gene. It was a rare form of congenital adrenal hyperplasia with an estimated incidence of about 1:50,000. The clinical manifestation of 17OHD includes hypertension and hypokalemia due to excessive synthesis of mineralocorticoid precursors, undermasculinized external genitalia in 46, XY males, and primary amenorrhea in 46, XX females.

In the Chinese population, 17OHD is the second common subtype of congenital adrenal hyperplasia after 21OHD. The CYP17A1-Y329fs mutation caused the truncation of CYP17A1 protein to a 418 amino acid fragment. *In vivo* analysis indicated that this mutation abolished both 17a-hydroxylase and 17,20-lyase activity. According to previous studies, Y329fs mutation of CYP17A1 gene was unique for East Asia countries. In addition, decreased adrenal 17a-hydroxylase activity and altered adrenal gland reserve for steroid biosynthesis were revealed in the genotype-proven carriers of the CYP17A1-Y329fs mutation.^1^ So, it was necessary to perform carrier screening of CYP17A1-Y329fs mutation in the Chinese population.

In this study, we intended to investigate the incidence of CYP17A1-Y329fs mutation in China as well as in other places of the world. According to the incidence of this mutation in the population of different regions of China, the migration of the mutation was also inferred from the PGG.Han database.

Fourteen patients with 17OHD and ten carriers with heterozygous CYP17A1 gene mutation were recruited for this study. All the subjects carried Y329fs mutation. Six patients were homozygous for Y329fs mutation, and eight patients were heterozygous for Y329fs mutation (Table S1). Cases 1–12 were tested by Infinium Global Screening Array-24 v1.0 (GSA) BeadChip (Illumina) containing 642,824 markers. Cases 13–24 were tested by Infinium Asian Screening Array-24 v1.0 (ASA) BeadChip containing 659,184 markers. Sixteen single nucleotide polymorphisms (SNPs) (Table S2) were selected around the CYP17A1 gene. In cases 1–12, these sixteen SNPs were acquired by imputation. In cases 13–24, five SNPs were contained in ASA Beadchip, and the other eleven SNPs were acquired by imputation.

Our analyses focused on single-nucleotide variants (SNVs) in 37,998 samples from PGG.Han (The Han Chinese Genome Database and Analysis Platform; https://www.hanchinesegenomes.org).^2^ Information on converging, Han131, KGP, EGDP, and 1025 was shown in Table S3. In genetics, all Han samples were further divided into six fine-scale substructure regions of populations (10,054 Central China Han (CCH); 3634 Northwest Han (NWH); 928 Northeast Han (NEH); 2060 Southwest Han (SWH); 17,622 Southeast Han (SHE); and 3700 South Coast Han (SCH)). Detailed methods were shown in the supplementary materials & methods.

Frequencies of sixteen SNPs in EGDP, 1025, and KGP datasets around the world were shown in Figure 1A. We also showed the frequencies of sixteen SNPs in six fine-scale substructure regions of PGG.Han, CONVERGE, and HAN131 datasets around China (Fig. 1B). Allele frequencies of rs10883783 were the lowest among the different regions in the world and around China. These sixteen SNPs showed incomplete linkage in 17OHD cases. However, they were complete linkage in PGG.Han, CONVERGE, and Han131 datasets (Fig. S1).

**Figure 1:**
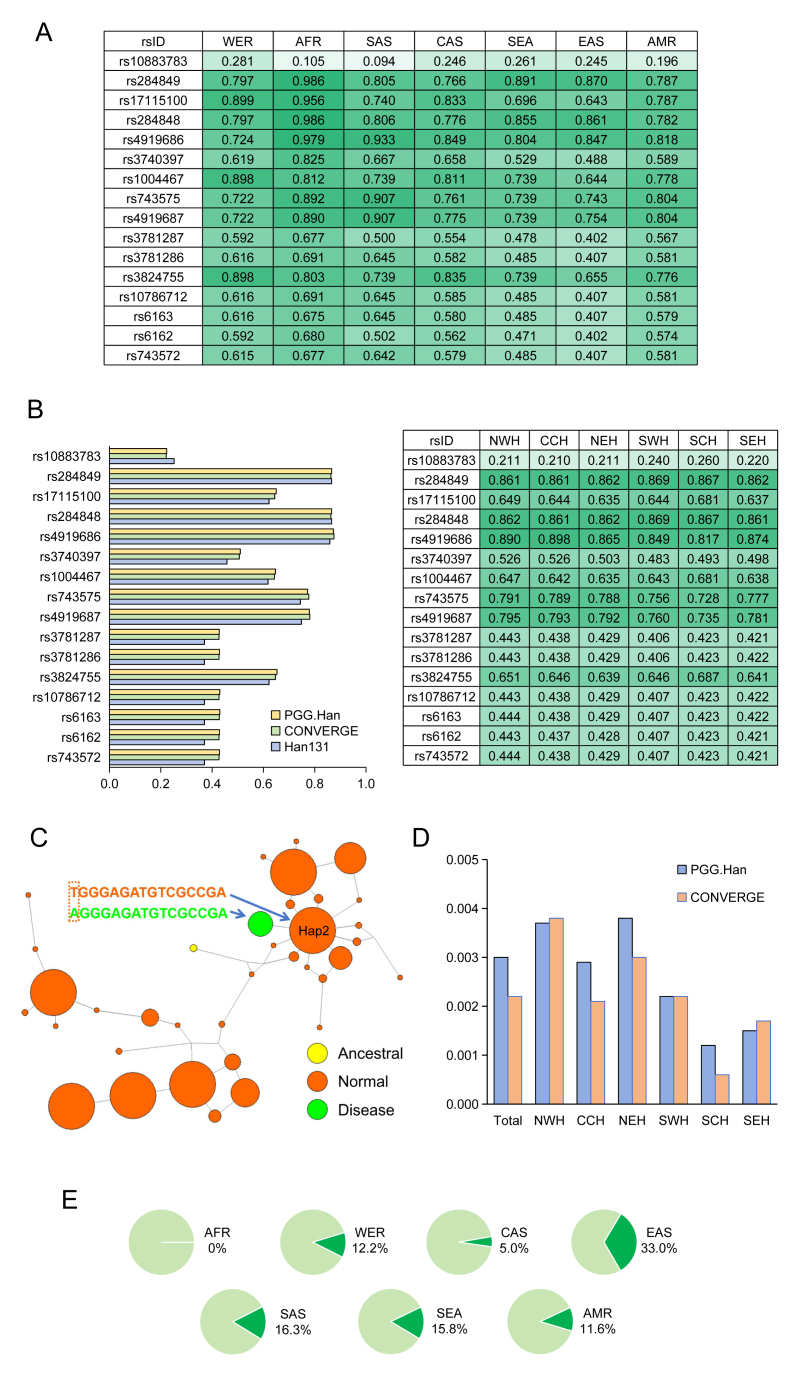
Haplotype analysis of single nucleotide polymorphisms (SNPs) in CYP17A1. **(A)** Frequencies of sixteen SNPs in EGDP, 1025, and KGP datasets around the world. **(B)** Frequencies of sixteen SNPs in six fine-scale substructure regions of China. **(C)** Origin of affected haplotype. **(D)** Prevalence of affected haplotype in PGG.Han and CONVERGE datasets in six substructure regions of China. **(E)** Prevalence of haplotype 2 around the world.

Haplotype network analysis is widely used for detecting the relationships among DNA sequences within a population. The affected haplotype (AGGGAGATGTCGCCGA) only has one different nucleotide from haplotype 2, which was the second-largest haplotype in the Chinese population. Together with indel mutation (CYP17A1-Y329fs), “A” allele in the affected haplotype came from haplotype 2 (Fig. 1C). In the PGG.Han dataset, the prevalence of affected haplotype ranged from 1.2‰ in SCH to 3.8‰ in NEH. However, in the CONVERGE dataset, the prevalence of affected haplotype ranged from 0.6‰ in SCH to 3.8‰ in NWH (Fig. 1D).

Previously, carrier screening was conducted by Sanger sequencing of suspected pathogenic genes in the overall population or patient's family members. For the first time, we introduced genome-wide association studies (GWAS) into carrier screening. Due to the reduced price of GWAS chips (less than $30 per person), it has become affordable for normal individuals to undergo a GWAS test. Moreover, GWAS chips could provide more information than Sanger sequencing, which means it had higher cost-effectiveness. According to our research, the carrier rate of CYP17A1-Y329fs was relatively higher in the northern population. So, the GWAS test could be included in premarital testing in these regions.

Separated by the Yangtze River, the Han Chinese people could be divided into two groups, northern Han and southern Han.^3^ There were three waves of massive population migrations from north to south due to warfare and famine in historical records. Investigation of genetic variation in both the non-recombining region of the Y chromosome (NRY) and mitochondrial DNA (mtDNA) in Han populations indicated the southward expansion of Han culture involves the mass movement of people.^4^ Due to the haplotype being inherited as a whole in the population, the migration of the population can be observed through common mutations. The affected haplotype of CYP17A1-Y329FS mutation also showed a clinal geographic pattern from north to south. So, aside from the Y chromosome and mtDNA, a rare mutation linked to haplotype might be another marker used to study migration pathway as well as demic diffusion model of the population.

The prevalence of haplotype 2 (TGGGAGATGTCGCCGA) was 33.0% in East Asian, subsequently 16.3% in South Asian, 15.8% in Southeast Asian, 12.2% in Western European and North American, 11.6% in Ad Mixed American, 5% in Central Asian, and 0% in African (Fig. 1E). The carrying rate of haplotype 2 in East Asians was higher than in other regions around the world in EGDP, 1025, and KGP datasets. However, haplotype 2 was not found in Africans. So, the difference between affected haplotype and haplotype 2 appeared separately when coming out of Africans. The highest carrying rate of haplotype 2 might be underlying the affected haplotype and was mainly found in East Asians. Except in the Chinese population, the affected haplotype was only found in ITU from KGP and Vietnamese south EGDP, respectively, which also indicated the East Asian origin of the affected haplotype.

In our study, most of the SNP data was interpolated. The accuracy of genotype imputation is influenced by several key factors: i) the sample size, the number of SNVs, and quality of the reference panel^5^; ii) ancestry consistency between the imputed samples and the reference population^5^; and iii) the allele frequency of the imputed SNV. In this study, we utilized Chinese samples, with the imputed disease samples sharing the same ancestry background as the reference panel. The reference panel comprises a large-scale cohort of ∼100,000 samples and over 8 million SNVs, ensuring the high quality of the imputation. Additionally, the SNVs investigated in this study are all common variants. Previous studies have shown that the imputation error rate for common variants is generally higher than that for rare variants. Therefore, we believe that the results of genotype imputation are reliable.

There were also limitations in our study. First, only 24 subjects carrying CYP17A1-Y329fs mutation were included in this study. The sample size was relatively small. Second, the affected haplotype was a rare haplotype that was only detected in East Asian populations. So, we cannot compare the incidence of affected haplotype with other global populations.

In conclusion, the carriers of CYP17A1-Y329fs mutation could be screened by population genetics in PGG.Han dataset. It will improve our knowledge of genetic counseling. Furthermore, the migration path of the population can be inferred by analyzing the frequency of CYP17A1-Y329fs mutation carriers in different regions of China.

## CRediT authorship contribution statement

**Bing Han:** Writing – review & editing, Writing – original draft, Funding acquisition, Conceptualization. **Xiaoxi Zhang:** Validation, Software, Methodology, Investigation, Formal analysis. **Chang Liu:** Validation, Methodology, Formal analysis. **Hui Zhu:** Methodology, Data curation. **Wenjiao Zhu:** Resources, Data curation. **Yue Xu:** Resources, Data curation. **Fengxue Zhang:** Resources, Data curation. **Kaiwen Zhang:** Resources, Data curation. **Shuhua Xu:** Supervision, Resources, Conceptualization. **Jie Qiao:** Supervision, Conceptualization.

## Ethics declaration

This study was approved by the ethics committee of Shanghai Ninth People's Hospital affiliated to Shanghai Jiaotong University School of Medicine (approval number 2017-437-T333). Written consent was obtained from all participants.

## Funding

This work was supported by the National Natural Science Foundation of China (No. 82470820), Innovative Research Team of High-level Local Universities in Shanghai (China) (No. SHSMU-ZDCX20212501), Cross-disciplinary Research Fund of Shanghai Ninth People's Hospital, Shanghai Jiao Tong University School of Medicine (China) (No. JYJC202412), Natural Science Foundation of Shanghai (China) (No. 22ZR1436600), and Clinical Research Program of 9th People's Hospital affiliated to Shanghai Jiao Tong University School of Medicine (China) (No. JYLJ202307).

## Conflict of interests

The authors declared no conflict of interests.

## Figures and Tables

**figs1:**