# Supplementary material for: Population-based screening of CYP17A1 Y329fs mutation carriers in the Han Chinese population
Source: Genes Dis. 2025 Apr 15;12(6):101647. doi: 10.1016/j.gendis.2025.101647 (PMC12275968; doi:10.1016/j.gendis.2025.101647)
Supplement: Multimedia component 1 [file mmc1.docx]

**Supplementary materials & methods**

For disease cohort dataset, SNVs with high missing rates were filtered out. We performed haplotype phasing using SHAPEIT4 ^1^. Then apply the IMPUTE2 ^2^ software for genotype imputation, using the PGG.Han ^3^ reference panel. We grouped the global populations by continent and compared the frequency distribution of the SNVs in the focused region. Using the substructure division of the Han Chinese population from previous studies ^3^, we compared the frequency distribution of these SNVs within different Han Chinese subgroups. Additionally, we used the low-coverage sequencing dataset CONVERGE and 131 deep sequencing samples to compare the distribution differences of these SNVs across different Han Chinese datasets. We constructed a median-joining haplotype network ^4^ for these SNVs in the 131 samples. The ancestral allele of each SNV was determined based on the ancestral sequences released by the 1000 Genomes Project.

[1] Delaneau O, Zagury JF, Robinson MR, Marchini JL, Dermitzakis ET. Accurate, scalable and integrative haplotype estimation. Nat Commun. 2019;10(1):5436.

[2] Howie BN, Donnelly P, Marchini J. A flexible and accurate genotype imputation method for the next generation of genome-wide association studies. PLoS Genet. 2009;5(6):e1000529.

[3] Gao Y, Zhang C, Yuan L, Ling Y, Wang X, Liu C, Pan Y, Zhang X, Ma X, Wang Y, Lu Y, Yuan K, Ye W, Qian J, Chang H, Cao R, Yang X, Ma L, Ju Y, Dai L, Tang Y; Han100K Initiative; Zhang G, Xu S. PGG.Han: the Han Chinese genome database and analysis platform. Nucleic Acids Res. 2020;48(D1):D971-D976.

[4] Bandelt HJ, Forster P, Röhl A. Median-joining networks for inferring intraspecific phylogenies. Mol Biol Evol. 1999;16(1):37-48.
