# Supplementary material for: Population-based screening of CYP17A1 Y329fs mutation carriers in the Han Chinese population
Source: Genes Dis. 2025 Apr 15;12(6):101647. doi: 10.1016/j.gendis.2025.101647 (PMC12275968; doi:10.1016/j.gendis.2025.101647)
Supplement: Multimedia component 2 [file mmc2.docx]

**Table S1**. Information of 16 SNPs.

| SNP | Location | Chr. position | Nucleotide Change |
| --- | --- | --- | --- |
| rs743572 | Exon 1 | 104597152 | T>C |
| rs6162 | Exon 1 | 104596981 | C>T |
| rs6163 | Exon 1 | 104596924 | G>T |
| rs10786712 | Intron 1 | 104596396 | G>A |
| rs3824755 | Intron 1 | 104595849 | C>G |
| rs3781286 | Intron 1 | 104595719 | G>A |
| rs3781287 | Intron 1 | 104595420 | A>C |
| rs4919687 | Intron 1 | 104595248 | C>T |
| rs743575 | Intron 2 | 104594906 | A>C |
| rs1004467 | Intron 3 | 104594507 | T>C |
| rs3740397 | Intron 5 | 104592675 | C>G |
| rs4919686 | Intron 6 | 104592249 | T>G |
| rs284848 | Intron 6 | 104592125 | C>T |
| rs17115100 | Intron 6 | 104591393 | C>A |
| rs284849 | Intron 7 | 104591182 | C>A |
| rs10883783 | Intron 7 | 104591152 | A>T |
| c.985_987 | Exon 6 | 104592420 | TAC>AA |

**Table S2**. Genotype of subjects with CYP17A1-Y329fs mutation.

| Subjects | Genotype |
| --- | --- |
| Case 1 | **p.Y329fs/p.Y329fs** |
| Case 2 | p.Y329fs /p.V311fs |
| Case 3 | p.Y329fs /WT |
| Case 4 | p.Y329fs /p.R96Q |
| Case 5 | **p.Y329fs/p.Y329fs** |
| Case 6 | p.Y329fs /WT |
| Case 7 | p.Y329fs /WT |
| Case 8 | p.Y329fs /c.1263G>A |
| Case 9 | p.Y329fs /WT |
| Case 10 | p.Y329fs /p.R239Q |
| Case 11 | p.Y329fs /WT |
| Case 12 | p.Y329fs /p.K144DEL |
| Case 13 | **p.Y329fs/p.Y329fs** |
| Case 14 | **p.Y329fs/p.Y329fs** |
| Case 15 | **p.Y329fs/p.Y329fs** |
| Case 16 | p.Y329fs/WT |
| Case 17 | p.Y329fs/WT |
| Case 18 | p.Y329fs/p.A421A |
| Case 19 | p.Y329fs/WT |
| Case 20 | **p.Y329fs/p.Y329fs** |
| Case 21 | p.Y329fs/WT |
| Case 22 | p.Y329fs/WT |
| Case 23 | Y329fs/Del D487-F489 |
| Case 24 | Y329fs/Del D487-F489 |

**Table S3**. Datasets information in this study.

| Datasets | Information |
| --- | --- |
| PGG.Han | Han, 81032 individuals |
| Converge | Han, 10640 individuals |
| Han131 | Han, 131 individuals |
| KGP | 26 populations, 2504 individuals |
| EGDP | 127 populations, 402 individuals |
| 1025 | 10 populations, 887 individuals |
